# STING-Activating Nanoparticles Combined with PD-1/PD-L1 Blockade: A Synergistic Approach in Cancer Immunotherapy

**DOI:** 10.3390/biomedicines13092160

**Published:** 2025-09-04

**Authors:** Dorota Bartusik-Aebisher, Kacper Rogóż, David Aebisher

**Affiliations:** 1 Department of Biochemistry and General Chemistry, Faculty of Medicine, Collegium Medicum, Rzeszów University, 35-310 Rzeszów, Poland; dbartusikaebisher@ur.edu.pl; 2 English Division Science Club, Faculty of Medicine, Collegium Medicum, Rzeszów University, 35-310 Rzeszów, Poland; kr117626@stud.ur.edu.pl; 3 Department of Photomedicine and Physical Chemistry, Faculty of Medicine, Collegium Medicum, Rzeszów University, 35-310 Rzeszów, Poland

**Keywords:** cGAS-STING, STING, cGAMP, PD-1, PD-L1, nanoparticles, combination therapy, immunogenic cell death, ICD

## Abstract

**Objectives**: Immunotherapy combining agonists of the cyclic GMP-AMP synthase–stimulator of interferon genes (cGAS-STING) pathway with PD-1/PD-L1 blockade shows promising preclinical results, although in clinical practice, it faces pharmacokinetic barriers, systemic toxicity, and an immunosuppressive tumor microenvironment (TME). Recent advances in and expansion of the cGAS-STING pathway as a therapeutic target have further highlighted its central role in innate and adaptive immune activation. The aim of this paper is to review combination strategies of STING and PD-1/PD-L1 checkpoint blockade therapies, triple-therapy strategies using a third component such as chemotherapy, radiotherapy, photodynamic therapy (PDT), and others, and the use of nanoparticles as carriers for these drugs. **Methods**: Reports in the literature on the mechanisms of STING + PD-1/PD-L1 synergy, as well as with the use of a third component and delivery systems, were analyzed. Current challenges and limitations, as well as prospects for the development of these therapies, are noted. **Results**: Activation of the cGAS-STING synergizes with blocking the PD-1/PD-L1 axis. The addition of a third component further enhances the anti-tumor effect through a stronger induction of immunogenic cell death (ICD), increased production of interferons and pro-inflammatory cytokines, repolarization of macrophages, and enhanced infiltration of T lymphocytes. **Conclusions**: Therapy with STING agonists and PD-1/PD-L1 checkpoint inhibitors, supported by nanotechnology vehicles and using a third therapeutic component, overcomes key pharmacological and immunological limitations. This multimodal immunotherapeutic strategy holds high translational promise, offering more effective and safer solutions in cancer immunotherapy.

## 1. Introduction

The immune system ensures the elimination of pathogens and the control of tumor cell growth. One of the key mechanisms initiating the immune response is the detection of foreign or damaged DNA in the cytoplasm of cells—a process mainly activated by the cGAS-STING (cyclic GMP-AMP synthase–stimulator of interferon genes) pathway. In recent years, this pathway has become the subject of intensive research as a potential therapeutic target in the immunotherapy of cancer and autoimmune diseases [1,2,3].

Activation of cGAS-STING induces the expression of type I interferons and pro-inflammatory cytokines, leading to the recruitment of immune cells. In the context of cancer, this pathway promotes the recognition and elimination of cancer cells, particularly in response to DNA-damaging therapies such as radiotherapy or chemotherapy [4,5]. cGAS-STING pathway is a central sensor of cytosolic double-stranded DNA (dsDNA), which can arise from viral infection, genomic instability, or cellular damage. Upon binding to dsDNA, cGAS catalyzes the production of cyclic GMP-AMP (cGAMP), which activates the adaptor protein STING located on the endoplasmic reticulum. STING then initiates downstream signaling through TBK1 and IRF3, leading to type I interferon (IFN-I) secretion and NF-κB activation. This response bridges innate and adaptive immunity by enhancing dendritic cell maturation and priming cytotoxic CD8+ T cells [6,7].

Beyond antiviral defense, the cGAS–STING pathway has emerged as a crucial mediator of anti-tumor immunity. Its activation can increase tumor antigen presentation, promote infiltration of effector T lymphocytes, and synergize with immune checkpoint inhibitors such as PD-1/PD-L1 blockade; however, excessive or chronic stimulation may also trigger immunosuppressive effects, including PD-L1 upregulation and regulatory T-cell expansion. These dual roles highlight both the therapeutic potential and the challenges of targeting this pathway in cancer immunotherapy [8,9].

A growing number of studies indicate that combining STING agonists with anti-PD-1/PD-L1 therapy may enhance treatment efficacy by synergistically stimulating the immune response [10,11]. In addition, the use of nanotechnology enables precise delivery of STING agonists to the site of action, reducing systemic toxicity and improving their pharmacokinetics and bioavailability [12,13]. This article discusses the mechanism of action of the cGAS-STING pathway, anti-PD-L1 immunotherapy, and their association in the context of nanoparticle use.

**Figure 1:**
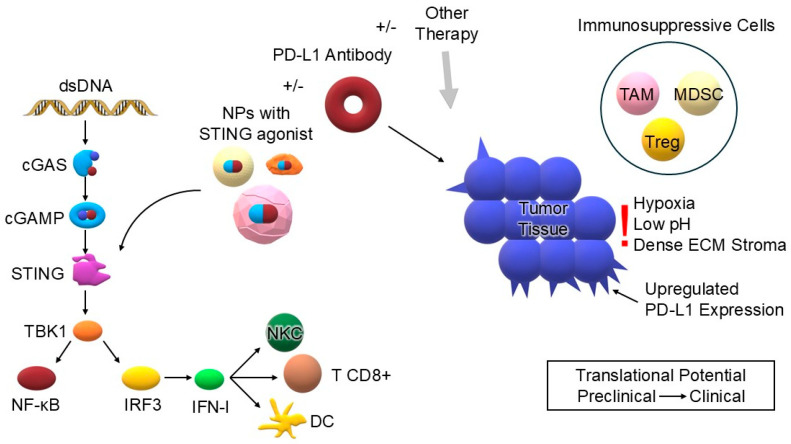
Conceptual framework and novelty of this review. The symbol +/− means that two therapies can be combined together or used separate. Cytosolic dsDNA activates the cGAS–STING pathway (dsDNA → cGAS → cGAMP → STING → TBK1 → IRF3/NF-κB), inducing IFN-I and pro-inflammatory signaling that primes dendritic cells (DC) and promotes cytotoxic CD8^+^ T-cell and NK-cell responses. A nanoparticle (NP) formulation carrying a STING agonist is shown targeting STING to enhance on-target activation while reducing systemic exposure. ±PD-1/PD-L1 antibody and ±other therapy (e.g., chemo/radiotherapy, PDT/SDT, nucleic acids, vaccines) feed into the tumor compartment to potentiate T-cell effector function and enable synergistic anti-tumor immunity. The tumor microenvironment (TME) features immunosuppressive cells (TAM, MDSC, Treg), hypoxia, low pH, and dense ECM stroma, which impede drug delivery and immune activity; upregulated PD-L1 expression on tumor cells illustrates potential adaptive resistance upon sustained STING signaling. Translational potential highlights the path from preclinical → clinical evaluation. Abbreviations: dsDNA—double-stranded DNA; cGAS—cyclic GMP–AMP synthase; cGAMP—cyclic GMP–AMP; STING—stimulator of interferon genes; TBK1—TANK-binding kinase 1; IRF3—interferon regulatory factor 3; NF-κB—nuclear factor kappa-B; IFN-I—type I interferons; DC—dendritic cell; CD8+ T—cytotoxic CD8+ T lymphocyte; NKC—natural killer cell; NPs—nanoparticles; PD-1/PD-L1—programmed cell death-1/programmed death-ligand 1; TAM—tumor-associated macrophage; MDSC—myeloid-derived suppressor cell; Treg—regulatory T cell; ECM—extracellular matrix (own elaboration).

To integrate the key concepts of this review, Figure 1 provides a one-page schematic of our conceptual framework and novelty: nanoparticle (NP)-mediated delivery of STING agonists directly targets the cGAS–STING axis and is combined with PD-1/PD-L1 blockade (±other adjuvant therapies) to elicit synergistic anti-tumor immunity. The figure also situates this strategy within the tumor microenvironment (TME)—highlighting dominant barriers (hypoxia, low pH, dense ECM, and immunosuppressive cells) and the potential for PD-L1 upregulation—and maps the translational pathway from preclinical studies to early clinical development.

## 2. Materials and Methods

This article is a narrative review of the literature. In the first step, publications containing the phrase “STING AND PD-L1 AND nanoparticles” were retrieved, which yielded a total of 94 results—44 from PubMed and 50 from Web of Science. After removing duplicates, 70 unique papers were left for further analysis. In a second step, the search was extended to include related terms such as follows: “STING pathway”, “PD-L1 immunotherapy”, “STING AND nanoparticles”, and the like. The aim of the second stage was to expand the scope of this review to include reports not included in the first stage but relevant to STING agonist and PD-1/PD-L1 inhibitor therapy.

**Figure 2:**
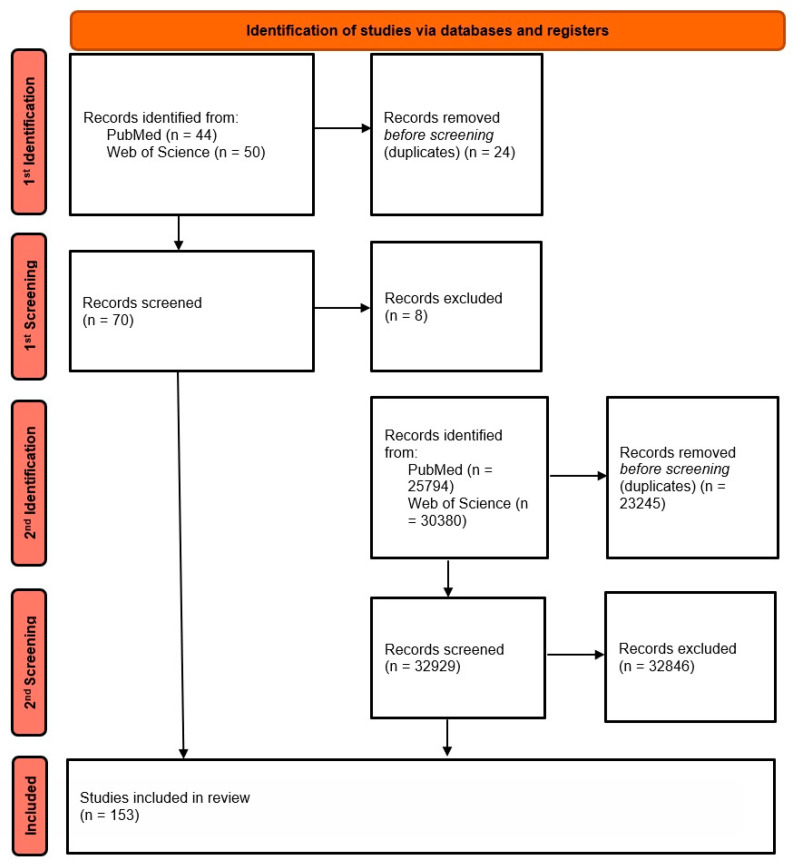
The literature selection process (PRISMA-style adapted flowchart for narrative review). The letter “n” state for the word “number”. (own elaboration).

After applying the inclusion criteria—mainly original clinical studies (prospective and retrospective), meta-analyses, and publication in English in the last ~5 years—and excluding papers not directly related to the topic, 153 publications were finally selected for detailed analysis. Figure 2 presents the literature selection process using an adapted PRISMA-style flowchart, tailored to the narrative review methodology.

## 3. The STING Pathway—Mechanism and Importance in the Immune Response

**Figure 3:**
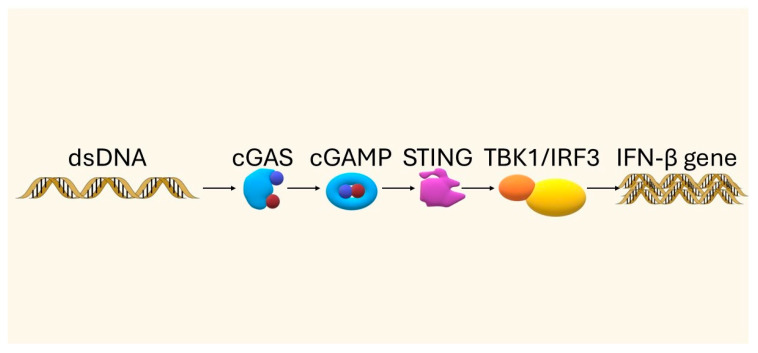
Simplified schematic of the cGAS–STING pathway, showing recognition of cytosolic DNA by cGAS, production of cGAMP, activation of STING, and downstream induction of type I interferons and pro-inflammatory cytokines (own elaboration).

The STING pathway has been briefly introduced in the Introduction Section; here, we provide a more detailed discussion of its mechanism and immunological relevance. The cGAS-STING pathway plays a key role in the body’s immune response by detecting the presence of double-stranded DNA (dsDNA) in the cytoplasm, which is usually a signal of infection or cell damage. Under physiological conditions, DNA outside the cell nucleus is eliminated by exonucleases such as TREX1 or RNase H2, although when homeostasis is disturbed—e.g., due to mitochondrial damage, the presence of retroviruses, genome instability, cell death, or viral infection—dsDNA accumulation in the cytoplasm occurs [6]. The presence of this DNA activates cGAS, the enzyme that catalyzes the synthesis of the cGAMP molecule from ATP and GTP. The cGAMP acts as a secondary messenger, binding to the STING protein located on the endoplasmic reticulum membrane, which initiates a further signaling cascade [6,7]. Upon activation, STING changes conformation and translocates to the Golgi apparatus, where it undergoes palmitoylation—a modification necessary for further signal transduction. This is followed by recruitment and activation of the kinase TBK1, which phosphorylates the transcription factor IRF3. Phosphorylated IRF3 dimerizes and translocates to the cell nucleus, where it induces gene expression, including the IFNB1 gene encoding type I interferon (IFN-β). In parallel, NF-κB pathways—both canonical (involving p65/p50) and non-canonical (involving p52/RELB)—are activated, further enhancing the inflammatory response [6]. Secreted type I interferons stimulate IFNAR receptors, activating the JAK-STAT pathway, which not only leads to the expression of antiviral genes but also enhances the activity of cytotoxic CD8+ T lymphocytes and antigen-presenting dendritic cells [6,14]. To provide a clear overview of the main steps of the cGAS–STING signaling cascade, a simplified schematic diagram is presented below (Figure 3). A more detailed mechanistic representation of the pathway in the context of PD-1/PD-L1 blockade is provided later in Section 6. Please refer to this figure when STING activation is mentioned later.

The importance of the cGAS-STING pathway extends beyond the antiviral response—it has an important role in recognizing and fighting cancer cells. For example, activation of cGAS-STING can be triggered by DNA-damaging therapies such as chemotherapy (cisplatin and doxorubicin) or radiotherapy, leading to the induction of an anti-tumor immune response. cGAS-STING also affects the expression of MHC class I molecules in tumor cells, increasing their recognition by cytotoxic lymphocytes [7]. Additionally, this pathway can be activated by the p53 protein—a key tumor suppressor—through TREX1 degradation and cytoplasmic DNA accumulation, demonstrating its involvement in both cellular and systemic mechanisms of tumor control [15]. Research into the therapeutic use of the cGAS-STING pathway is also not insignificant. Intensive work is underway on agonists of this pathway, especially cyclic dinucleotides (CDNs), which have potential as vaccine adjuvants or components of anticancer therapies. Their efficacy is enhanced when used concomitantly with immune checkpoint inhibitors (CPIs), which may lead to a synergistic therapeutic effect [7,14]. In clinical trials, the first STING agonist evaluated in humans was a synthetic cGAMP analog known as ADU-S100 (MIW815). When administered topically to the tumor, it induced systemic activation of CD8+ T cells, confirming its immunostimulatory effects, although on its own, its therapeutic efficacy was limited [16]. Other STING agonists in clinical trials include MK-1454, E7766, BMS-986301, BI-1387446, TAK-676, SNX281, HG-381, and GSK3745417, among others. Their mechanism of action is to mimic natural cyclic dinucleotides such as cGAMP [17].

Activation of the cGAS-STING pathway plays a key role in the induction of the anti-tumor response through production of type I interferons, maturation of dendritic cells (DCs), and recruitment of cytotoxic T cells (CD8+). However, chronic stimulation of this pathway may lead to immunosuppressive effects by increasing PD-L1 levels and activation of regulatory (Treg) lymphocytes, which may favor tumor growth [18]. Excessive activation of the pathway can lead not only to chronic inflammation but also to autoimmune diseases [7,14]. In addition, environmental factors within the tumor, such as hypoxia, oxidative stress, or changes in lipid and glucose metabolism, modulate cGAS-STING activity [18]. Hence, research on cGAS-STING inhibitors and safe drug delivery systems is as important as research on agonists [7,14].

In summary, the cGAS-STING pathway is a key component of the immune system that links innate and acquired responses. Its role in detecting cellular abnormalities and triggering the immune response makes it an attractive therapeutic target for the treatment of cancer as well as infectious and autoimmune diseases [6,7,14,19].

## 4. Anti-PD-L1 Immunotherapy—Limitations and the Need to Enhance Action

**Figure 4:**
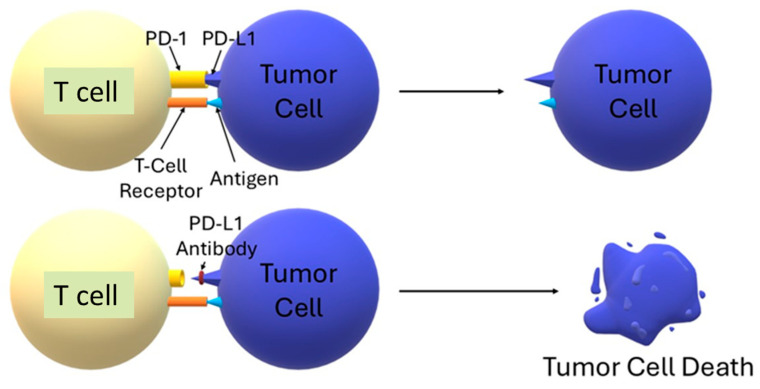
The role of the PD-1/PD-L1 pathway in inhibiting T cell activation. Binding of the PD-1 receptor on the surface of the T lymphocyte to the PD-L1 ligand expressed by the tumor cell results in inhibition of the immune response, even with simultaneous recognition of the antigen by the TCR receptor. Blockade of PD-1 or PD-L1 interrupts this inhibitory signal, allowing activation of effector T cell function and leading to elimination of the tumor cell [20,21,22].

The PD-1/PD-L1 axis is a key mechanism for controlling T-cell activity, maintaining immune homeostasis, and at the same time, it is sometimes used by tumors to escape immune surveillance. The PD-1 receptor, which is found on the surface of T cells, upon engagement by its ligand PD-L1—often overexpressed on cancer cells—recruits SHP2 phosphatases via ITSM motifs, leading to the inhibition of TCR signaling and inhibition of cytokine secretion and effector lymphocyte proliferation. This results in T-cell exhaustion and apoptosis, as well as an increase in Treg cell populations, which together impair the body’s ability to destroy tumor cells [20]. Blocking this pathway with antibodies against PD-1 or PD-L1 restores T-cell function, allowing them to reactivate and eliminate the tumor [21,22]. To illustrate the immunosuppressive mechanism of action of the PD-1/PD-L1 axis and the effect of its blockade in anticancer therapy, a simplified diagram of signaling occurring on the surface of T cells and tumor cells is shown below (Figure 4).

Examples of approved antibodies blocking the PD-1/PD-L1 pathway include pembrolizumab and nivolumab (anti-PD-1) as well as atezolizumab, durvalumab, and avelumab (anti-PD-L1) [22]. Immunotherapy based on the blockade of checkpoints such as PD-1/PD-L1 has revolutionized the treatment of certain cancers, such as melanoma, non-small-cell lung cancer, bladder cancer, and kidney cancer [23]. However, its efficacy is often limited by low levels of T-cell infiltration and insufficient inhibitor binding to PD-L1 in the tumor environment [24]. Therapeutic response is achieved by only 20–40% of patients, and many initial responders develop subsequent resistance [25,26]. Consequently, regimens combining anti-PD-1/PD-L1 therapy with other interventions—including chemotherapy, radiotherapy, angiogenesis inhibitors, microbiota modulation, and STING pathway agonists—are increasingly being used in the clinic [25,27,28,29,30].

## 5. Nanoparticles as Carriers of STING Agonists

To overcome the pharmacokinetic and pharmacodynamic limitations of STING agonists, a range of nanocarriers has been developed. Modern delivery systems for STING agonists enhance their therapeutic efficacy. The nanocarriers improve the stability and bioavailability of molecules such as cyclic nucleotides (CDNs) or non-CDN compounds, and their composition circumvents inherent limitations such as short half-life or non-selective activity [18,31]. For example, encapsulating STING’s natural ligand, 2′3′-cGAMP, within the polymerosome structure significantly prolonged its half-life and increased its penetration into the tumor microenvironment (TME). Compared to free cGAMP, which exhibits an extremely short half-life (~2 min) and negligible tumor accumulation, STING-NPs markedly improve pharmacokinetics and therapeutic efficacy. Encapsulation prolonged the elimination half-life to 1.3 h (a ~40-fold increase) and enhanced systemic exposure (AUC) by 6.5-fold. Unlike free cGAMP, which failed to accumulate in tumors, STING-NPs delivered 1–3% of the injected dose to B16-F10 tumors within 4 h. This resulted in robust STING pathway activation, with serum IFNβ levels reaching ~15,000 pg/mL at 4 h, alongside >20-fold increases in intratumoral CD4+ and CD8+ T cells, effectively converting “cold” tumors into inflamed microenvironments. Therapeutically, STING-NPs reduced tumor burden by ~75% in B16-F10 melanoma and ~80% in E0771 breast cancer, extending survival by 26–33 days, whereas free cGAMP showed negligible activity. These findings provide quantitative evidence that nanoparticle-based delivery overcomes the pharmacokinetic limitations of free agonists and enables potent, tumor-selective STING activation with durable anti-tumor responses [32]. The use of nanoparticles allows for the delivery of more than one substance, allowing for a synergistic, enhanced effect of therapy. For example, a novel immunotherapy strategy for pancreatic cancer based on immunostimulating nanoparticles (immuno-NPs) overcomes the highly immunosuppressive tumor environment by simultaneously activating two pathways of innate immunity—STING and TLR4. Dual-agonist immuno-NPs (MPLA^high/cdGMP) induced ~11-fold and ~22-fold higher IFNβ production than cdGMP-NPs or MPLA^high-NPs, respectively. In vivo, ~56% of DCs, 79% of macrophages, and 52% of NK cells in the tumor microenvironment internalized NPs within 24 h, while free agonists distributed broadly, with disproportionately higher uptake in the liver and spleen. This translated into excessive systemic immune activation (e.g., ~28-fold increase in hepatic DCs with free agonists vs. ~11.5-fold intratumoral increase in NPs). Therapeutically, MPLA^high/cdGMP-NPs reduced tumor bioluminescence by ~94% and extended survival, with ~14% of mice remaining tumor-free at day 100, whereas free agonists failed to prevent relapse. Thus, NP delivery ensures preferential intratumoral accumulation, limits systemic toxicity, and improves therapeutic outcomes [33].

Nanocarriers are crucial for the efficient delivery of cGAS-STING pathway agonists, which are inherently negatively charged, hydrophilic, and rapidly degradable molecules, such as cyclic dinucleotides (CDNs) [34,35]. For this reason, different carrier platforms have been developed.

### 5.1. Lipid Nanoparticles (LNPs, Liposomes)

Lipid nanoparticles (LNPs) provide efficient delivery of STING agonists, their controlled release, reduced toxicity, and are relatively easy to produce on a large scale. The typical LNP structure contains ionizable lipids, phospholipids, cholesterol, and PEG-lipids, which ensure stability, immune system protection, optimal dissolution, and long circulation time [36,37]. Among the LNPs, we can find traditional liposomes loaded with, for example, cGAMPs [38]. In addition, we can distinguish lipid–calcium-phosphate nanobubbles (LCP-NPs), which show high encapsulation efficiency, gradual release, and strong activation of the STING pathway [39]. Another example is lipid nanodiscs with PEG-lipids conjugated to cyclic dinucleotides (CDN–PEG-lipids). They show better penetration into tumors than traditional liposomes, and a single dose effectively induces immune memory in an animal model [40].

### 5.2. Polymeric Nanocarriers

Polymer carriers are created from biodegradable materials, e.g., β-amino ester polymers, polysiloxanes, often modified with linkers that are sensitive to enzymes or pH parameters, allowing for selective release of CDNs in target cells. They often have the ability to penetrate biological barriers and are able to precisely reach the tumor site [41,42]. Polymeric nanoparticles show greater stability, minimal toxic effects, and controlled release inside the cytosol, resulting in a stronger immune response [43]. Examples of polymers and nanoparticles used in STING-agonist therapy include PLGA polymer and ONP-302 nanoparticles [44], or epirubicin-conjugated glycopolymer (EPI) in the form of mB4S nanocarrier [45].

**Figure 5:**
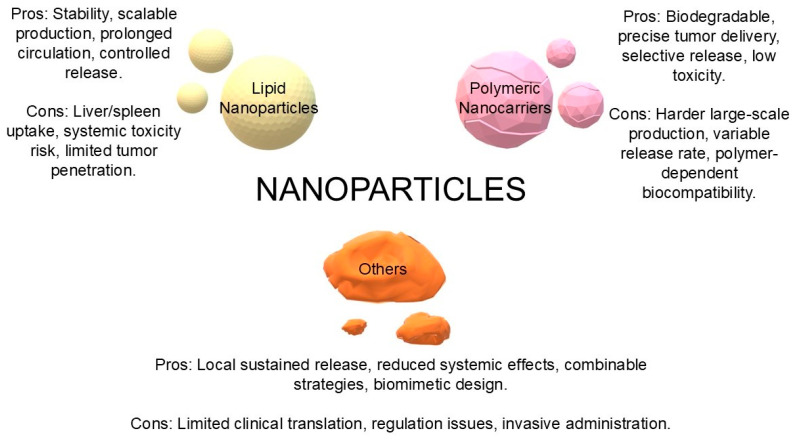
Comparative advantages and limitations of different nanocarrier platforms (LNPs, polymeric nanocarriers, and others) for the delivery of STING agonists in cancer immunotherapy (own elaboration).

Other systems, such as hydrogels, microspheres, protein adjuvants, or, for example, inorganic nanoparticles with a biomimetic surface, such as M@αM [46,47,48]. Many show promise in implantable mediators or in local release of STING agonists with minimal toxicity and local control. To facilitate the comparison of different STING agonist delivery systems, we have summarized graphically their main advantages and limitations. The nanoplatforms presented below—lipid (LNP), polymeric, and other novel systems (hydrogels, microspheres, and inorganic nanoparticles)—differ in terms of stability, toxicity, controlled-release capabilities, and translational potential. A graphical comparison (Figure 5) provides a better understanding of their strengths and challenges in the context of future clinical applications.

Key physicochemical properties affecting the immunoactivity of nanoparticles include the following: (1) size and surface charge—nanoparticles with a size of 50–150 nm optimize the Enhanced Permeability and Retention (EPR) effect, and the slightly positive charge facilitates internalization and escape from the endosome; (2) stability and protection of agonists—encapsulation protects against enzymatic degradation and increases bioavailability; (3) stimulus-dependent release—pH, reductase, and lysosomal enzymes allow for the release of agonists in target cells and minimize systemic effects; (4) surface modifications—cell-targeting ligands or PEGylation improve biodistribution and reduce immune agility [40,49,50,51]. The final choice depends on the intended therapeutic use and the desired pharmacokinetic and immunostimulatory profile.

## 6. Combination of STING Agonists and Anti-PD-1/PD-L1 Therapy

### 6.1. Mechanism of Synergy

**Figure 6:**
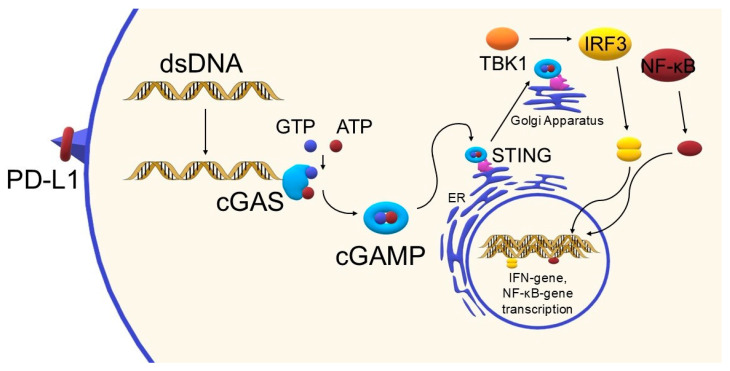
Schematic of the cGAS-STING signaling pathway and its interaction with PD-L1 blockade. Double-stranded DNA (dsDNA) of cytoplasmic origin (e.g., from cancer cells, damaged mitochondria, or viruses) activates the enzyme cGAS, which, with the participation of ATP and GTP, synthesizes the secondary messenger cGAMP. cGAMP binds to the STING protein located in the membrane of the endoplasmic reticulum (ER), which initiates its translocation to the Golgi apparatus and activation of the kinase TBK1. TBK1 phosphorylates the factor IRF3, which dimerizes and, together with NF-κB, translocates to the cell nucleus, inducing the expression of type I interferons (IFN-I) and pro-inflammatory cytokines. Activation of this pathway can lead to a potent anti-tumor response. Simultaneous blockade of PD-L1, seen in the figure, prevents immunosuppression, allowing more efficient elimination of tumor cells in combination therapy [10,45].

The synergy between the cGAS-STING pathway agonist and PD-1/PD-L1 blockade is based on an enhancement of the anti-tumor response. STING agonists trigger innate immune activation (type I IFN production) that enhances antigen presentation and effector T-cell recruitment; combining this with PD-1/PD-L1 blockade restores effector function (see Section 3 and Figure 3) [10,45]. Figure 6 shows the mechanism of activation of the cGAS-STING pathway, a key component of the type I immune response and an important target in modern anticancer therapies, especially in combination with checkpoint immunotherapy.

In addition, in target cells, the STING agonist can induce immunogenic death (ICD) by releasing DAMPs (damage-associated molecular patterns) such as ATP, increasing calreticulin expression, and thereby enhancing antigen presentation by dendritic cells. As a result, the immunological influx of CD8+ T cells into the tumor is intensified and PD-L1 blockade protects them from inhibiting the “stop” signal. By increasing the number of mature DCs and CD8+ T cells in the tumor, and decreasing the presence of suppressive MDSCs, the tumor microenvironment is transformed from immunosuppressive to immunoactive [45]. It is also known that activation of the cGAS-STING pathway and simultaneous suppression of PD-L1 immunosuppressive factors effectively overcomes tumor resistance to radiotherapy and immunotherapy [52,53]. It is important to remember that the effectiveness of immune therapies may depend not only on the agents used but also on the regimen in which they are administered. In a model of melanoma metastasis to the lung, it was shown that only two or three doses of lipid nanoparticles containing a STING pathway agonist (STING-LNP) with an anti-PD-1 antibody, administered at two-day intervals, enable activation of the immune response and potentiate the effect of the anti-PD-1 antibody. Optimization of the therapy schedule may, therefore, significantly affect its anti-tumor effect [54].

### 6.2. Lipid and Polymeric Nanocarriers

The use of lipid nanoparticles (STING-LNPs) containing an agonist of the STING pathway enabled effective activation of NK cells in a model of metastatic pulmonary melanoma (B16-F10) refractory to anti-PD-1 therapy. The combination of STING-LNP with anti-PD-1 showed a synergistic anti-tumor effect, even in tumors lacking effective antigen presentation. These results indicate that targeting NK cells through activation of the STING pathway may be a promising strategy for overcoming resistance to cancer immunotherapy [10]. A synergistic effect of therapy using nanoparticles containing a STING pathway agonist (cGAMP) with mannose alongside anti-PD-L1 antibodies was shown by Li et al. The combination therapy showed a significantly better anti-tumor effect in melanoma treatment than each drug used alone, highlighting the potential of this strategy [55]. A nanoparticle-based 2′3′-cGAMP delivery system (STING-NPs) was also developed that activates the STING pathway directly in neuroblastoma (NB) cancer cells. Activation of the STING pathway also showed a synergistic effect with anti-PD-L1 therapy [56]. Physically cross-linked hyaluronan-lipid nanoparticles (HLHCs) containing cGAMP effectively overcome the limitations associated with their bioavailability and transport into the cytoplasm of antigen-presenting cells. HLHCs provide prolonged circulation in the body, controlled release, and high efficiency of STING pathway activation in cancer cells. Even a single dose induced a strong anti-tumor response and regression of advanced tumors in MC38 and B16F10 models, especially when combined with anti-PD-L1 therapy [57]. Another example is the immunomodulatory PLGA nanoparticles (ONP-302), which have proven to be an effective tool in activating the anti-tumor response by reprogramming myeloid cells. ONP-302 does not deliver an exogenous drug or agonist within itself but acts as a stand-alone immunomodulator. Carboxylated PLGAs primarily target macrophages and other myeloid cells in the spleen and tumor microenvironment. Acting through the cGAS-STING pathway and an IL-15-dependent mechanism to activate NK cells and CD8+ lymphocytes, ONP-302 reduced tumor growth in models based on classical immunotherapy. Furthermore, ONP-302 treatment led to an increase in PD-1/PD-L1 expression in the tumor environment, which enabled the effective use of checkpoint inhibitors (anti-PD-1) [44].

### 6.3. Biomimetic Platforms

In response to low levels of T-cell infiltration and insufficient inhibitor binding to PD-L1 in the immunosuppressive tumor microenvironment (TME), a novel PEC nanosystem—a hybrid structure composed of E. coli and tumor cell membranes—was developed to enable selective delivery of PD-L1 inhibitors directly to the tumor. Under TME-specific oxidative stress, the PEC was remodeled into a structure with an increased density of PD-L1-binding proteins, enabling more efficient blockade of this immunosuppressive pathway and PD-L1 degradation in lysosomes. In addition, the presence of the cGAS-STING activator (HZD) promotes innate immunity (see Section 3/Figure 3) [24]. Another mB4S nanocarrier system is based on a branched glycopolymer conjugated to epirubicin (EPI) and containing a STING agonist (diABZI). The whole product was coated on the 4T1 tumor cell membrane to facilitate targeted delivery. EPI, released in response to acidic tumor pH, induces immunogenic cell death. Simultaneously, diABZI activates the cGAS-STING pathway. In both the 4T1 (breast cancer) and CT26 (colorectal cancer) models, the mB4S system significantly converted the TME from immunosuppressive to immunoactive. Combination therapy with anti-PD-L1 antibodies showed a synergistic anti-tumor effect, offering a promising strategy for the treatment of tumors with low immunogenicity [45].

### 6.4. Manganese as a STING Agonist

Manganese ions are also used in therapy aimed at stimulating the STING pathway. Manganese oxides (MONs) provide an innovative platform to support cancer immunotherapy through multifaceted action within the tumor microenvironment. They are able to generate oxygen, reduce PD-L1 expression, convert macrophages to a pro-inflammatory form, and activate the cGAS-STING pathway. In this way, they enhance the immune response, improving the efficacy of therapies such as checkpoint immunotherapy, photodynamic therapy (PDT), or cancer vaccines [58]. The BM@MnP-BSA-aPD-1 membrane-coated microglia nanoplatform was designed to cross the blood–brain barrier and target the immunosuppressive microenvironment of glioblastoma multiforme (GBM). Once it reaches the tumor, it activates the cGAS-STING pathway, induces ICD, and simultaneously blocks the PD-1/PD-L1 axis due to the presence of the aPD-1 antibody. In addition, the photothermal (PTT) effect enhances the immune response, leading to TME remodeling and a cascade of anti-tumor immune activation [59]. Another nanoplatform, TPP-MMONs, based on a manganese-enriched mesoporous silica carrier and targeting mitochondria through the TPP ligand, takes advantage of the high concentration of glutathione in cancer cells for the controlled release of Mn^2+^. These ions induce mitochondrial stress, leading to a loss of mitochondrial membrane potential, an increase in mtROS, and the opening of mPTPs, resulting in the release of mitochondrial DNA (mtDNA) into the cytoplasm. mtDNA, in combination with Mn^2+^ as a direct activator of cGAS, effectively initiates cGAS-STING [60]. Furthermore, in a novel approach to immunotherapy of triple-negative breast cancer (TNBC), biosynthetic Bio-MnSe nanoparticles were designed using Chlorella vulgaris as a bioreactor. Despite their very low manganese content, Bio-MnSe exhibited potent immunomodulatory properties—effectively inducing reactive oxygen species (ROS) production and activation of the cGAS-STING pathway (see Section 3/Figure 3). The combination of Bio-MnSe with a checkpoint inhibitor (aPD-L1) further potentiated the therapeutic effect [61]. The smart nanosystem (B&V@ZB-MCL), on the other hand, combines three components: losartan for collagen barrier breakdown, a STING agonist (Vadimezan), and a PD-L1 inhibitor (BMS-1). This strategy not only facilitates the penetration of drugs and immune cells into the tumor but also enhances the immune response and inhibits tumor escape mechanisms from treatment. This therapy led to apparent collagen degradation, an influx of immune cells, and a reduction in tumor mass [62]. In the treatment of TNBC, the combination of the previously described Bio-MnSe nanoparticle with doxorubicin (Dox) as an ICD inducer, also proved effective, resulting in a Bio-MnSe@Dox formulation that not only directly eliminated tumor cells but also activated dendritic cells and initiated a potent tumor-specific response [61]. Another interesting solution is the DPGMA platform, based on dendrimers and a metal–phenol network (MPN), which allows simultaneous delivery of an anti-PD-L1 antibody, an anticancer drug (gossypol), and manganese ions (Mn^2+^). In the tumor environment, this complex induces immunogenic tumor cell death by chemotherapy and oxidative stress generated by Mn^2+^-mediated redox reactions. DPGMA integrates three mechanisms of immunotherapy, ICD, STING activation, and immune checkpoint blockade, while enabling MRI imaging, offering a comprehensive and targeted therapeutic approach [63]. Engineered RGD-MnFe2O4 nanoparticles, combining MRI imaging functions with STING pathway activation, enable selective delivery of manganese to the tumor while limiting its toxicity. The combination of these nanoparticles with anti-PD-L1 therapy enhanced the immune response in various tumor types, confirming the potential of this strategy [64]. These results indicate the high efficacy of biogenic nanocarrier strategies in breaking the immunosuppressive TME and enhancing the immunotherapeutic response in TNBC. Finally, Mn@SCD1and@αPD-L1 nanoparticles induce a “ferroptosis storm” and activation of the cGAS-STING pathway by releasing three key components: Mn^2+^, which enhances manganese; an SCD1 inhibitor, which inhibits lipid metabolism; and αPD-L1, which blocks PD-L1. The simultaneous regulation of lipid metabolism and inhibition of the nt/β-catenin pathway promotes the infiltration of immune cells into the tumor, offering a promising strategy for the treatment of so-called “cold” tumors [65]. The encapsulation of Zn^2+^ ions in addition to Mn^2+^ ions in the nanoparticle further enhances cGAS-STING activation. Zn^2+^ ions initiate degradation of the mutant p53 protein (mutp53) via the ubiquitination pathway, abolishing its inhibitory effect on immunity [66].

## 7. Other Combinations Using STING Agonists and Anti-PD-L1 Therapy

In this section, we have summarized the main approaches for combining STING agonists and anti-PD-1/PD-L1 therapy with other therapies. The inclusion of an additional, third component—from chemotherapy, radiotherapy, and PDT/SDT to nucleic acid delivery platforms, cuproptosis, or nanovaccines—allows not only additional damage to the tumor but also enhances the immune response and breaks down resistance. The mechanism of action of the different types of third component therapy, examples of nanoparticles used in the study, cancer model, and main results are summarized in Table 1.

### 7.1. Chemotherapy

Augmenting STING-agonist plus anti-PD-1/PD-L1 therapy with a chemotherapeutic agent represents a promising strategy for the treatment of immunotherapy-resistant cancers. Numerous studies have demonstrated that nanocarriers facilitate co-delivery of cytotoxic and immunomodulatory agents, resulting in synergistic anti-tumor activity. One example is the PhenPt NP nanoparticle system, based on the reduction-sensitive polymer PHHM, which enables controlled delivery of the cationic platinum drug PhenPt. In addition to its cytotoxic effect, it activates the cGAS-STING pathway, inducing an innate response (see Section 3/Figure 3). Combination with anti-PD-L1 therapy resulted in inhibition of tumor growth and immune response, also in metastases [67]. A similar approach was used in the NP2 system, containing a cisplatin prodrug (Pt(IV)) and a WEE1 kinase inhibitor (MK1775). In response to glutathione, bladder cancer tumor cells are activated, leading to DNA damage and strong activation of the STING pathway [68]. To increase the efficacy of immunotherapy in lung cancer, the STACI platform, based on Pdx-NP™ nanoparticles co-delivering the STING activator, anti-PD-L1 antibody, and volasertib, a cytostatic drug, was developed. The use of STACI led to remodeling of the tumor microenvironment and improved survival without signs of toxicity [69]. The innovative design was applied to G-M NPs, a minimalist nanoplatform containing the STING agonist (MSA-2) and gemcitabine. These molecules induced immunogenic cell death and activation of the cGAS-STING pathway. Their combination with anti-PD-L1 therapy enabled almost complete inhibition of TNBC tumor growth and protection against recurrence [70]. Another approach uses RMP@Cap nanocapsules containing mitoxantrone, anti-PD-L1 antibodies, and zinc phenolic complexes. Induced pyroptosis led to the release of mtDNA and strong activation of STING, while the use of an erythrocyte membrane envelope increased the circulation time of the nanoparticles and their accumulation in TNBCs [71]. In response to the limitations associated with the cardiotoxicity of doxorubicin (Dox), the PMDDH system was developed, integrating Dox and metformin in a hyaluronan-based nanocarrier. The combined action of Dox on DNA and AMPK activation by metformin enhanced anti-tumor efficacy and reduced systemic toxicity while enhancing STING and ICD activation [72]. Li et al. developed a multicomponent acidic pH-responsive nanoparticle system that also delivered Dox, a peptide that blocks the PD-1/PD-L1 pathway, as well as a TLR4 agonist (MPLA). Its design was enriched with the immunomodulatory polymer PEG-PC7A. Therapy led to enhanced release of ICD signals, as well as activation of the STING and TLR4 pathways. A mouse model showed significant inhibition of osteosarcoma tumor growth and induction of immune memory [73]. An integrated therapy has also been developed in the form of a three-component nanosystem, combining the gemcitabine prodrug, STING agonist, and anti-PD-L1 antibody; this system effectively inhibited the growth of immunotherapy-resistant tumors while inducing long-term immune memory [74]. PPD nanoparticles containing DMXAA were also contracted. STING activation in combination with anti-PD-L1 therapy led to complete eradication of both the primary focus and metastatic lesions [75]. In response to the limited efficacy of immunotherapy in patients with head and neck squamous cell carcinoma (HNSCC), innovative C-SNPs nanoparticles delivering shikonin (SHK), a natural inducer of ICD, were designed. SHK not only induced tumor necrosis but also interfered with DNA repair mechanisms in tumor cells, activating the cGAS-STING pathway and increasing PD-L1 expression. The combination of C-SNPs with anti-PD-1 therapy increased the infiltration of dendritic cells and CD8+ lymphocytes, although the therapeutic response was moderate. Enrichment of nanoparticles with Mn^2+^ ions (C-SMNPs) significantly enhanced the immune response and tumor inhibition in vivo [76].

### 7.2. Radiotherapy

Researchers have shown that the combination of irinotecan (IRIN) with radiotherapy destroys colorectal cancer cells more effectively than either therapy used alone. The mechanism of this effect is based on activation of the cGAS/STING pathway, which initiates a strong immune response (see Section 3/Figure 3). In order to limit the toxicity of IRIN, the drug was encapsulated in special silicasome-type nanoparticles, which allowed its controlled release. This therapy has shown even greater efficacy in combination with anti-PD-1 immunotherapy, making it a promising strategy for preoperative neoadjuvant therapy (NCRT) in colorectal cancer patients [77].

### 7.3. PDT

Photodynamic therapy (PDT) uses a light-activated photosensitizer to generate reactive oxygen species (ROS) that damage mitochondria, other organelles, and DNA of cancer cells, inducing immunogenic cell death and the release of DAMPs, which promotes the recruitment and activation of dendritic cells and T lymphocytes [78,79]. Tian et al. developed a novel therapeutic molecule, BMA, which combines activation of the STING pathway, reprogramming of glutamine metabolism, and photodynamic damage to mitochondria. Such a complex mechanism leads to a profound remodeling of the immunosuppressive TME. BMA, through continuous free radical production, promotes dendritic cell maturation, enhances antigen presentation, and induces a strong inflammatory response. Importantly, the released tumor genetic material acts as a personalized cancer vaccine. The therapy effectively transforms “cold” tumors into “hot” tumors, significantly enhancing the efficacy of immunotherapy with anti-PD-L1 antibodies and inducing an abluminal effect. This strategy offers a promising solution for overcoming resistance to cancer immunotherapy [80]. An innovative approach to synergistic anticancer therapy was the use of TPP-Ce6@siPD-L1 nanoparticles, in which the photosensitizer Ce6 and siRNA against PD-L1 self-assemble into stable nanoparticles without classical carriers (carrier-free). The TPP-Ce6 complex provides targeting to the mitochondria of cancer cells, where ROS are generated upon irradiation, leading to mitochondrial damage, activation of the cGAS-STING pathway, and activation of ICD. Simultaneous delivery of siPD-L1 enables effective silencing of PD-L1 expression, unblocking CD8+ lymphocyte function, which in breast cancer models led to robust tumor regression [81]. An analogous TCe6@Cu/TP5 construct enriched with copper ions (Cu^2+^) and the immunomodulatory peptide TP5 promoted the innate immunity in a glioblastoma multiforme (GBM) model. After PDT, cuproptosis and degradation of PD-L1 by AMPK are induced. TCe6@Cu/TP5 nanoparticles effectively penetrate the blood–brain barrier via a copper transport mechanism and have demonstrated in vivo GBM efficacy [82]. Other nanoparticles, MP@PPS NPs, also release a photosensitizer and STING agonist in response to oxidative stress in the tumor environment, initiating apoptosis and ICD. Due to their small size, MP@PPS NPs have a high permeability into the tumor and a prolonged circulation time. Combining this therapy with PD-L1 blockade further enhanced the therapeutic effect and reduced the risk of tumor recurrence [83]. Innovative nanomotors with an asymmetric structure show the ability to simultaneously activate the cGAS-STING pathway and block the PD-1/PD-L1 checkpoint, representing a promising strategy for breast cancer immunotherapy. Driven by bubbles generated from the catalytic reaction of platinum with hydrogen peroxide, the nanoparticles efficiently penetrate tumor tissue. Internally, they contain the photosensitizer Ce6, which, upon irradiation, induces DNA damage and triggers an immune response via the cGAS-STING pathway. At the same time, the CLP002 peptide blocks the PD-1/PD-L1 interaction. With their ability for targeted delivery, movement, and bioimaging, these nanomotors offer a multidirectional and precise intervention in the tumor microenvironment [84]. Multi-target activation of the immune system was also achieved by a system based on porous H-MnO_2_ nanoparticles capable of controlled release of DNAzyme, Mn^2+^ ions, chlorin e6, and glycyrrhetinic acid (GA). The released DNAzyme specifically degrades PD-L1 mRNA, and Mn^2+^ activates the cGAS-STING pathway (see Section 3/Figure 3). At the same time, Ce6 and GA induce ICD under the influence of light, enhancing the therapeutic effect. This system offers coordinated immune activation and effective inhibition of metastasis [85].

### 7.4. SDT

As part of new strategies based on STING pathway activation, biomimetic M@αM nanoadjuvants have been developed, combining diagnostic, immunomodulatory, and therapeutic functions. Under ultrasound, M@αM induce immunogenic tumor cell death in melanoma and breast cancer by releasing Mn2+ ions and activating the cGAS-STING pathway. At the same time, inhibition of amino acid transport by α-MT leads to a glutamine deficit in the tumor microenvironment, further enhancing the immune response. This therapy enhances the maturation of DCs and effectively inhibits tumor growth, recurrence, and lung metastasis, especially when combined with a PD-L1 inhibitor. An additional advantage is that the treatment can be monitored due to the photoacoustic and resonance properties [46]. Ultrasensitive Ce6/PTX Nbs nanobubbles have also been developed, combining sonodynamic therapy, chemotherapy, and immunotherapy. The nanoparticles contain chlorin e6 (Ce6) and paclitaxel (PTX), and through modification with RGD peptides, precisely target cancer cells. When activated by ultrasound, they induce ICD, activate cGAS-STING, and enhance CD8+ lymphocyte infiltration. In the 4T1 model, they have shown high anti-tumor efficacy, especially in combination with PD-L1 blockade, with low toxicity and the possibility of simultaneous imaging [86].

### 7.5. Provision of Nucleic Acids

In response to the limited efficacy of immunotherapy in low-immunogenic tumors, a number of advanced nucleic-acid-based nanoplatforms have been developed that activate the cGAS-STING pathway (see Section 3/Figure 3). One example is the AHA@MnP/siPD-L1 nanomodulator, which binds siRNA against PD-L1 and Mn^2+^ released in acidic TMEs. Manganese ions activate the STING pathway, while siRNA abrogates lymphocyte inhibition by PD-L1. This therapy reduces Treg cell and M2 macrophage populations, promoting a pro-inflammatory environment and demonstrating high anti-tumor efficacy in a triple-negative breast cancer model [87]. Another strategy is the TT-LDCP nanoparticles, which deliver siRNAs that block PD-L1 and encode interleukin 2 (IL-2) to increase T-lymphocyte activity. The use of thymine-modified dendrimers enhances the immunoadjuvant effect by activating the STING pathway, while limiting systemic toxicity [88]. Another approach is the cGAMP-siPDL1@GalNPs platforms, which combine a STING agonist with siRNA against PD-L1. With galactose as the targeting ligand, the nanoparticles selectively target cells overexpressing GLUT-1 and galectin transporters, releasing cargo when exposed to the tumor environment and laser light [89]. Microneedles containing copper–zinc sulfides and siPD-L1 have also been shown to activate STING via cuproptosis and ICD, while blocking immunosuppression and improving TME oxygenation, which inhibited melanoma growth and metastasis [90]. dsDNA nanocarriers (dsDNA@DMONs) are also a promising approach, activating STING directly within the tumor and leading to near-complete regression in animal models, especially when combined with the PD-L1 inhibitor [91]. Even more advanced systems, such as H/LDz-M@B, self-synthesize dsDNA in response to microRNAs present in tumor cells, enabling simultaneous activation of the innate and adaptive responses, elimination of metastasis, and long-term immunity [92]. Also of note was the plant-derived nanoplatform GP@DMX NV, isolated from the roots of Glycyrrhiza uralensis and functionalized with DMXAA and an anti-PD-L1 antibody. These nanoparticles enabled the delivery of miRNAs (miR2916) and bioactive metabolites to cancer cells, inducing apoptosis and activation of the STING pathway [11].

### 7.6. Cuproptosis

Cuproptosis is a newly discovered copper-dependent cell death mechanism that has found application in cancer immunotherapy through induction of mitochondrial stress and activation of the cGAS-STING pathway. TNBC therapy uses a bimetallic Cu-ZnO2@PDA nanoplatform (CZP NPs), which in the acidic tumor environment, releases Cu^2+^ and Zn^2+^ ions as well as hydrogen peroxide, generating ROS and damaging mitochondria. This leads to the release of mtDNA and irreversible cuproptosis. The synergy of mtDNA and Zn^2+^ results in a strong activation of the cGAS-STING pathway and an increase in PD-L1 expression, allowing PD-L1 inhibitors to work more effectively. In addition, NIR laser irradiation enhances the anti-tumor effect. The combination of CZP NPs with anti-PD-L1 therapy led to a strong immune response and inhibition of tumor growth [93]. A similar mechanism has been used in CLDCu nanoparticles designed to treat pulmonary metastases. Based on a chitosan–copper shell and a core of disulfiram and heparin, these particles release Cu^2+^ and DSF in the acidic tumor microenvironment to form a toxic CuET complex, inducing cuproptosis. At the same time, activation of the STING pathway stimulates an immune response. Inhalation of CLDCu in animal models led to a marked inhibition of pulmonary metastasis and, in combination with anti-PD-L1 therapy, generated a strong synergistic anti-tumor response [94].

### 7.7. Nanovaccines

Combining radiofrequency ablation (RFA) with a nanovaccine that activates the STING pathway may be a promising therapeutic approach in liver cancer. In preclinical studies, layered double-hydroxide (LDH) nanoparticles carrying cGAMP and adsorbing tumor antigens released during RFA were used. Such a platform effectively delivered cGAMP to tumor cells and the immune system, enhancing the type I interferon response and promoting dendritic cell maturation. Infiltration of cytotoxic lymphocytes within the tumor and lymph nodes was also increased. Importantly, the nanovaccine significantly improved the efficacy of anti-PD-L1 therapy, indicating a strong synergistic effect of both strategies [95]. A similar approach was used in the construction of an acid-reactive polymeric nanovaccine containing both a STING agonist and a defined neoantigen. In the acidic environment of endosomes, the selective release of antigen and simultaneous activation of STING occurred. In B16-OVA melanoma and 4T1 breast cancer models, this therapy showed clear anti-tumor effects, especially when combined with the PD-L1 inhibitor [96]. These results indicate the potential of nanovaccines as a tool for the simultaneous presentation of tumor antigens and activation of innate immunity, offering a new direction in personalized cancer immunotherapy.

### 7.8. Other Approaches

In recent years, advanced strategies have emerged that combine chemodynamic therapy (CDT) or photothermal therapy (PTT) with activation of the cGAS-STING pathway and blockade of the PD-1/PD-L1 axis, allowing for multi-targeted stimulation of anti-tumor immunity. Genetically modified membrane-coated nanozymes (cCM@MnAu) induce a cascade response in the tumor microenvironment. These nanozymes generate toxic hydroxyl radicals, enhancing the effect of chemodynamic therapy and inducing immunogenic tumor cell death. Simultaneously released manganese ions activate the STING pathway. This approach enables efficient blockade of PD-1/PD-L1 checkpoints and leads to a potent immune system response against primary tumors and metastases, as demonstrated in breast cancer models [97]. In the work of Zhu et al., a peroxidase enzyme-based nanozyme (POD-like nanozyme) was developed that induces mitochondrial damage and mitochondrial DNA (mtDNA) release by generating rROS, activating the cGAS-STING pathway in cancer cells. The resulting PNEC conjugate, containing a nanozyme conjugated to a peptide that blocks PD-L1 via an MMP-2-sensitive linker, exhibits selective anti-tumor activity and the ability to promote transcellular transport of mtDNA and its uptake by dendritic cells. Due to its positive surface charge, the nanozyme can effectively adsorb mtDNA, potentiating the maturation of DCs and activation of CD8+ lymphocytes [98]. As part of a novel “subtractive strategy”, biomimetic CISP (cholesterol-insufficient PD-1 T-cell-membrane-coated particles) nanoparticles were also designed, in which the removal of cholesterol from the T-cell membrane significantly improved pharmacokinetic properties without compromising biological function. The membrane-preserving PD-1 expression effectively blocked the immunosuppressive PD-1/PD-L1 axis, which was activated secondarily by the STING agonist (SR-717), allowing full activation of the T-lymphocyte response. At the same time, the delivery of the photothermal agent enabled local destruction of tumor cells through a heat effect. Removal of cholesterol reduced complement deposition and phagocytosis by monocytes by more than 50%, resulting in twice the accumulation in the tumor. In melanoma and colorectal tumor models, the system induced complete remissions in more than 80% and 40% of mice, respectively, while maintaining good bioleakage [99]. A complex but promising strategy for cancer therapy may be the use of nanoplatforms based on metal–organic structures (MOFs), such as MnxOy/(A/R)TiO_2_ (MTO), which enable simultaneous sonodynamic therapy (SDT), chemodynamic therapy (CDT), and immunotherapy. Upon reaching the tumor, MTO nanoparticles reduce glutathione levels and initiate the production of reactive oxygen species, leading to immunogenic tumor cell death. At the same time, the released Mn^2+^ ions activate the STING pathway in dendritic cells. Combining this strategy with anti-PD-L1 therapy shows a vaccine-like effect—enhancing anti-tumor immunity and providing long-term protection against metastasis and tumor recurrence [100]. Another promising avenue of research is the use of extracellular vesicles of plant origin as carriers for anticancer drugs. In one such approach, a Tf-GEVs@Pt(IV) nanocarrier was developed using grapefruit vesicles (GEVs) to deliver a triple-functional platinum(IV) compound to the tumor. Once in the tumor microenvironment, the nanoparticle released active ingredients responsible for DNA damage, reduction in inflammation, and degradation of fibrosis. At the same time, the cGAS/STING pathway was activated and PD-L1 expression was inhibited [101]. In order to overcome the limitations of modified Salmonella-based therapy, i.e., primarily the activation of neutrophils with a pro-cancerous N2 type, MnO_2_ nanoparticles activating the STING pathway were used to lead to the repolarization of neutrophils from the N2 to N1 phenotype. This overcomes the immunosuppressive tumor microenvironment and enables efficient infiltration and activation of CD8+ lymphocytes. Combination therapy with Salmonella and MnO_2_ resulted in complete inhibition of tumor growth and 80% survival at 40 days, confirming the potential of this strategy as a new form of anticancer therapy using targeted modulation of neutrophils [102]. In another study, in response to the limited efficacy of CAR-T therapy in solid tumors due to the immunosuppressive microenvironment, an innovative aPD-L1 NVs@cGAMP inhalation nanosystem was developed. These nanobubbles, containing a STING pathway agonist (cGAMP) and a fragment of an anti-PD-L1 antibody, enable the selective delivery of immunostimulatory cargo to tumor cells in the lung. Activation of the STING pathway induces an interferon response, leading to the elimination of suppressor cells and increased infiltration of CAR-T cells. At the same time, PD-L1 blockade prevents their functional exhaustion. Combination therapy with CAR-T and aPD-L1 NVs@cGAMP effectively inhibited lung cancer growth and metastasis in animal models [103]. The PSMP nanoparticle, constructed from cGAMP-containing PLGA@MnO_2_ nanoparticles and membrane-coated T cells with high PD-1 expression, effectively targets pre-metastatic niches (PMNs) in lung adenocarcinoma. Simultaneous blockade of PD-L1 on MDSCs and activation of cGAS-STING in PMNs overcomes immunosuppression and effectively prevents metastasis formation [104].

## 8. Clinical Data and Translational Aspects

Agonists of the cGAS-STING pathway are mainly being evaluated in early phase studies, such as monotherapy, or, more commonly, in combination with PD-1/PD-L1 inhibitors. Both CDN agents administered intratumorally (IT) and small-molecule non-CDN agonists with systemic administration (IV) are being tested. Clinical reports to date indicate clear pharmacodynamic signals (induction of interferon response genes and increase in concentrations of chemokines such as CXCL10), with limited anti-tumor activity in monotherapy. In combination with PD-1/PD-L1 inhibitors, isolated, sometimes durable responses have been described, although efficacy is heterogeneous between tumor types and treatment regimens [17,105,106,107]. The most commonly observed side effects are flu-like symptoms (fever, chills, fatigue), pain at the site of administration (for IT), and transient increases in inflammatory parameters. Severe immunological events and full-blown CRS cases are rare but possible, especially with systemic dosing and in combination with immune checkpoint inhibitors (ICIs). In clinical practice, it is important to gradually increase the dose, consider a pulsatile regimen (avoiding chronic STING activation) and local administration in selected indications, which may limit systemic toxicity [17,108,109]. IT administration achieves high drug concentrations in the tumor with less systemic exposure, although it is limited to anatomically accessible lesions and is more difficult in visceral tumors or microscopic spread. IV administration simplifies logistics and allows treatment of disseminated disease but is associated with rapid clearance, uptake by the reticuloendothelial system, and greater risk of AE.

Clinically, indications with low immunogenicity (“cold” tumors) and/or with low T-cell infiltration may be the most justifiable indications, where the excitation of the innate response has the greatest potential to transform TME [105,107]. Prospective biomarkers include the following: baseline cGAS-STING axis activity (cGAS/STING e-expression and ISG signatures); PD-L1 status; “T-inflamed” signatures; STING allelic variants with differential responsiveness; and negative regulators (e.g., ENPP1 and TREX1) that may suppress STING signaling. Prospective biopsy collection for pharmacodynamic assessment (p-TBK1/IRF3, ISG) and monitoring of serum chemokines (e.g., CXCL10), as rapid indicators of pathway activation, are recommended in clinical trials [5,110,111]. Interspecies differences and STING polymorphisms in humans limit the translatability of preclinical results. Additionally, chronic STING activation may increase PD-L1 expression and promote T-cell depletion, which clinically necessitates short, pulsed exposures and strict sequencing against ICI. Limiting systemic exposure (local administration, “site-restricted” systems) and designing carriers with stimulus-dependent release to minimize toxicity outside the tumor are also key [5,107,110]. Although most of the platforms described remain at the preclinical stage, their design advantages (selective release, co-delivery of STING agonist with ICI/other drug, and cellular targeting, reduced clearance) are consistent with translational needs. Clinical implementation, however, will require resolution of CMC and manufacturing scale issues, batch standardization, assessment of carrier immunogenicity (e.g., CARPA reactions), and post-tenancy accelerated PEG clearance for repeated administrations [108,112,113].

## 9. Challenges and Current Limitations

Despite promising preclinical results, the implementation of therapy combining STING agonists with PD-1/PD-L1 checkpoint inhibitors in clinical practice faces a number of significant barriers. Many small-molecule STING agonists (e.g., CDN) are rapidly cleared from the body and penetrate tumors poorly, forcing the use of high or frequent doses. This can lead to systemic inflammation and off-target toxicity [17,114,115]. The applied doses and dosages of selected nanoparticles described in this review are presented in Table 2.

**Figure 7:**
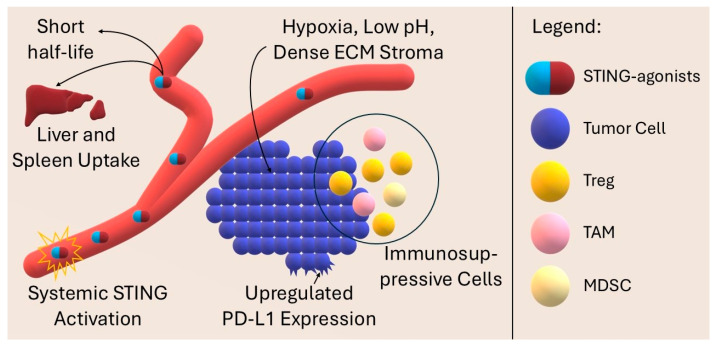
Main barriers limiting the efficacy of therapy combining STING agonists with PD-1/PD-L1 inhibitors. In the bloodstream: rapid clearance of STING agonists, their uptake by the liver and spleen, and the risk of systemic toxicity. In the tumor microenvironment (TME): Hypoxia, acidic pH, dense ECM stroma, and the presence of immunosuppressive cells (MDSCs, TAMs, Treg), which impede drug delivery and activation and favor increased PD-L1 expression [18,117,118,119,125,126,127,128,129].

In addition, non-specific uptake by the liver and spleen limits the concentration of the drug in the tumor, reducing the effectiveness of therapy [32]. Systemic activation of STING leads to strong production of type I interferons and pro-inflammatory cytokines, which can result in cytokine release syndrome (CRS), liver damage, and vascular leakage. Combination with checkpoint inhibitors may exacerbate immune-related side effects if doses are not appropriately adjusted and restricted to the site of action [116,117]. An immunosuppressive TME, which is characterized by hypoxia, acidic pH, and dense stroma, hinders the delivery and activation of both STING agonists and antibodies. The presence of myeloid-derived suppressor cells (MDSC), tumor-associated macrophages (TAM), and regulatory lymphocytes (Treg) may attenuate the pro-inflammatory effect of therapy [109,117,118,119]. Importantly, the composition and immunogenicity of the tumor microenvironment vary considerably between different cancer types, and even between patients with the same histological diagnosis. Some tumors are “hot” and infiltrated by T cells, while others are “cold” and largely devoid of adaptive immune activity. In addition, intrinsic defects in the STING pathway (e.g., epigenetic silencing and mutations) or variable PD-L1 expression further modulate therapeutic responses. This heterogeneity represents a major obstacle to generalizing the synergistic effects of STING activation and PD-1/PD-L1 blockade across cancer types [120,121]. Furthermore, although a single STING pulse can stimulate anti-tumor immunity, its prolonged activation can paradoxically increase PD-L1 expression and lead to T-cell depletion, promoting an immunosuppressive niche and even autoimmunity [117,122,123,124]. Figure 7 shows the key obstacles that impede the delivery of drugs to the site of action and limit their therapeutic efficacy and safety.

The effective use of STING therapy in combination with PD-1/PD-L1 inhibition in the treatment of cancer is limited by numerous pharmacological, immunological, and tumor microenvironment challenges, although novel drug delivery and combination strategies are being developed to overcome existing barriers and enhance the efficacy and safety of therapies. In clinical practice, special attention is required to monitor CRS and early markers of activation (fever, tachycardia, and increase in CRP/IL-6) and, in case of association with ICI, to manage irAE according to guidelines (steroid/anti-cytokine algorithms). Sequencing (e.g., short STING pulses followed by ICI) and the location of administration may limit the risk of side effects while maintaining synergistic effects [107,108].

From a safety perspective, the balance between efficacy and toxicity of novel STING-activating nanoplatforms remains particularly important. Preclinical data indicate that material modifications—such as PEGylation, biomimetic cell coatings, or biodegradable polymers—can reduce non-specific accumulation in the liver and spleen and limit systemic cytokine release [125,126,127,128,129]. In addition, the use of pH- or enzyme-responsive tumor systems improves selective delivery to the TME, reducing the risk of CRS and other side effects [130]. However, despite promising results, long-term toxicological and pharmacokinetic studies, which are essential for clinical translation and assessment of the real safety profile of the therapies in question, are lacking.

Multimodal strategies, such as combining nanoparticle-based delivery of STING agonists with checkpoint blockade and an additional therapeutic modality, have been specifically designed to mitigate these pharmacokinetic and toxicity-related challenges. A more detailed discussion of such advanced delivery approaches is provided in the subsequent Prospects and Future Directions Section.

## 10. Prospects and Future Directions

To overcome current limitations and optimize STING therapy in combination with PD-1/PD-L1 checkpoint blockade, intensive research in the area of advanced delivery systems is ongoing. As described previously, engineered pH- or enzyme-responsive nanoparticles enable selective release of STING agonists in the tumor microenvironment, increasing their local concentration and reducing systemic toxicity [114]. In addition, topical delivery systems such as microneedles or implantable hydrogels can limit the activation of STING in the tumor, minimizing side effects and ensuring high local concentrations [131,132,133]. It is also worth mentioning that artificial intelligence (AI) and machine learning are increasingly being used to design nanoparticles that deliver immunotherapeutics. AI enables prediction of biodistribution, optimization of size, surface area, and composition of carriers, as well as personalization under the patient’s genetic profile. Reviews in the literature indicate that learning algorithms are far superior to trial-and-error methodologies in optimizing nanocarriers for anticancer therapies [134,135]. In the STING agonist mRNA nanocarrier study, machine learning was used to optimize the design of LNP carriers, which significantly improved mRNA and cGAMP delivery and in vivo immune response [136]. AI supports the development of personalized therapies with high efficacy and minimal toxicity [137]. Considering the strong heterogeneity of the tumor microenvironment across cancers, future development of STING-based combination therapies will require stratification of patients according to tumor type, immune infiltration patterns, and STING pathway functionality. Integration of multi-omics approaches and biomarker discovery may help to identify subsets of patients most likely to benefit from this therapeutic synergy [120,121,138]. Incorporating gene expression profiling of the cGAS-STING pathway, PD-L1 status, and specific gene signatures associated with T-cell infiltration allows precise selection of patients who may best respond to therapy. For example, studies in patients with NSCLC have shown that higher cGAS expression may predict poorer efficacy of PD-1/PD-L1 inhibitors, even when PD-L1 expression is high, which is probably related to the immunosuppressive effect of TGF-β [139]. In contrast, analysis of TCGA data in colon cancer confirmed the correlation between expression of cGAS/STING components and PD-L1 expression, suggesting the need to consider both elements for predicting response [140]. In addition, different genetic variants of STING show differential responsiveness to STING fires and different prevalence in populations—which needs to be taken into account in the design of clinical trials [141]. To monitor the immune response and the risk of cytokine release syndrome (CRS) in real time during immunotherapy, a system based on DNA origami, a plasmonic nanoantenna capable of ultrasensitive detection of the cytokines TNF-α and IFN-γ by surface-enhanced Raman scattering (SERS), was developed. These nanoantennas use a logically controlled opening of the DNA structure and release of gold nanowires in response to cytokine binding to specific aptamers, which translates into a change in the optical signal. This technology allows simultaneous and programmable detection of multiple cytokines in serum, which has been validated in mouse models subjected to immunotherapy based on PD-1/PD-L1 inhibitors and STING agonists, offering a novel tool for precise monitoring of therapy and patient safety [142]. These multimodal delivery strategies, therefore, directly counteract the key pharmacokinetic and safety limitations described in the Introduction Section. Nanoparticle encapsulation protects agonists from rapid degradation and clearance, while stimuli-responsive or tumor-targeted systems enhance intratumoral accumulation and limit systemic exposure. In turn, localized approaches such as microneedles and hydrogels restrict STING activation to the tumor site, lowering the risk of systemic cytokine release and immune-related toxicity while maintaining synergistic efficacy in combination with PD-1/PD-L1 blockade [106,107,108,115,116,117].

A number of nanoparticles have passed the preclinical phase of trials, demonstrating a favorable safety profile. For example, ONM-501 has passed the preclinical phase. It showed a positive pharmacokinetic and tolerability profile, which supports its further development in cancer treatment [143]. However, clinical trials using encapsulated STING-agonist nanoparticles with/without association with anti-PD-1/PD-L1 therapy are still lacking. There are no results from phase I/II studies such as NCT04592484, which aimed to evaluate the dose, safety, and pharmacodynamic profile of STING-agonist-loaded exoSTING exosomes (CDK-002) in patients with solid tumors [144]. In contrast, there are several clinical trials of STING agonists alone with/without checkpoint blockade therapy without the use of nanoparticles, which appear promising for further studies. For example, IMSA101, a cGAMP analog, has passed clinical phase I. Analogs administered to the tumor showed an overall favorable safety profile. In a phase I study involving 22 patients, only one grade 1 adverse reaction (cytokine release syndrome) was reported at a dose of 800 µg, which resolved after one day. A grade 3 adverse reaction (arthropathy) occurred at a dose of 1200 µg. An amount of 1200 µg was set as the recommended dose for further study [145]. In total, 41 patients were treated with agonist BI 1387446 with or without the combination with ezabenlimab. Phase I results indicated good tolerability of the drug [146]. Similarly, another STING agonist, E7766, i.t. administered in a phase I/Ib study E7766, showed strong immune cell activation [147]. Ulevostinag is a STING agonist, which has shown anti-tumor activity in recurrent squamous cell carcinoma of the head and neck in a phase II study. In combination therapy with pembrolizumab, 4 out of 8 participants achieved a complete or partial response. The most common adverse effect among patients was fever [148]. Although clinical trials of STING-agonist nanoparticles are still lacking, their use in preclinical settings allows for better tumor penetration, reduced systemic toxicity, and potentially increased efficacy of PD-1/PD-L1 combination therapy. This indicates that nanoparticles represent a promising translational strategy and are a natural direction for further clinical research. Table 3 summarizes selected ongoing clinical trials combining STING agonists with PD-1/PD-L1 blockade in various cancer types.

Currently, there are no clinical studies that directly compare dual therapy (STING agonist + PD-1/PD-L1) with triple therapy (with the addition of chemo/RT/PDT/SDT/nucleic acids/cuproptosis/nanovaccines). Successful triple configurations are published in animal models but rarely are they juxtaposed directly with the corresponding dual therapy in the same paper and model. Current reviews emphasize the early stage of translation and the paucity of standardized comparisons (endpoints: biodistribution, T1/2, IFN-I/chemokines, tumor control, and toxicity), which limits the strength of the conclusions. Prospective comparative designs in standardized models are needed to resolve when the addition of a third module truly improves the benefit/risk ratio relative to well-optimized dual therapy [153].

## 11. Conclusions

Combination therapy of cGAS-STING pathway agonists with PD-1/PD-L1 axis blockade represents a novel and promising strategy in the fight against cancers resistant to traditional immunotherapy. Despite the challenges of the short half-life of STING small molecules, systemic toxicity and immunosuppressive TMEs, effective nanotechnological solutions are being developed. Lipiodine nanoparticles, polymeric and biomimetic platforms, supported by AI algorithms, enable selective drug delivery, controlled release of STING agonists and PD-1/PD-L1 inhibitors, and real-time monitoring of immune parameters. An interdisciplinary approach integrating advanced materials, precise biomarkers, and machine-learning algorithms can significantly enhance the efficacy and safety of therapy, paving the way for its implementation in clinical practice.

## Figures and Tables

**Table 1:** Summary of the type of additional therapy component, its mechanism of action, examples of nanoparticles, cancer model, and main result of the study [9,66,67,68,69,70,71,72,73,74,75,76,77,78,79,80,81,82,83,84,85,86,87,88,89,90,91,92,93] (→ means directon from substrate to the product.).

Additional Therapy	Mechanism of Action (Component 3)	Examples of Nanoparticles	Cancer Model	Main Result
Chemotherapy	Cytotoxic DNA damage in cancer cells	PhenPT NPs	A549 in vitro	Induction of apoptosis occurs by DNA damage, both with PhenPt NPs and with cisplatin. Cisplatin activates the canonical cGAS-STING pathway.
			LLC1 in vivo	PhenPT NPs show stronger cytotoxicity than cisplatin. PhenPt NPs in combination with anti-PD-L1 therapy induce a strong systemic immune response and inhibit the growth of tumor metastases.
		NP2 (Pt(IV) + MK1775)	Biu87, UMUC3, T24, Mb49 in vitro	NP2 showed a significantly higher cytotoxic effect and apoptosis rate than the other molecules compared. The best result was achieved in the T24 and Mb49 models.
			Biu87, Mb49 in vivo	NP2 demonstrates efficacy in vivo. The use of anti-PD-L1 therapy further enhanced the anti-tumor efficacy.
		STACI	LLC-JSP in vivo	Administration of volasertib, a STING agonist, and an anti-PD-L1 antibody in 3 doses increased median survival from 11 days to 24 days.
		G-M NPs	4T1 in vivo	The use of nanoparticles alone with the STING agonist and gemcitabine resulted in an 87.1% inhibition of tumor growth. Administration of an additional anti-PD-L1 antibody enhanced this effect, with a final inhibition of 98.0% of the primary tumor.
		RMP@Cap	4T1 in vivo	Administration of RMP@Cap induced tumor cell pyroptosis, which enhanced STING activation. Introduction of anti-PD-L1 into the nanocapsule attenuated the inhibitory effect of tumor cells on the recruitment of cytotoxic T cells. The erythrocyte membrane coating enabled a longer half-life and better accumulation of the drug in the tumor.
		PMDDH	MCF7, MDA-MB-231, 4T1 in vitro and in vivo	The use of the nanoplatform increased the therapeutic efficacy of doxorubicin and reduced its cardiotoxicity. In addition, the use of metformin resulted in activation of the AMPK pathway, decreased PD-L1 expression, and promoted ICD.
Radiotherapy	Induction of DNA breaks and ROS by radiation	IRIN-silicasomes	MC38 in vitro and in vivo	IRIN-silicasomes in combination with radiotherapy showed higher efficacy than irinotecan (IRIN) alone in combination with radiotherapy. Activation of the immune response and potentiation by anti-PD-1 therapy were confirmed.
Photodynamic Therapy (PDT)	Generation of ROS by photosensitizer (Ce6, BMA) leading to mitochondrial damage	TPP-Ce6@siPD-L1	4T1 in vitro and in vivo	The therapeutic potential of nanoparticles was demonstrated. The addition of photodynamic therapy as a third treatment component accelerated DC maturation, increased T-lymphocyte infiltration, enhancing the immune response. The biosafety profile was assessed as favorable.
		TCe6@Cu/TP5	GL261, U87 in vitro and in vivo	We combined therapy with STING agonists, PDT, and copper ions, activating cuproptosis, encapsulated in a single nanoparticle. Activation of the systemic immune response, the ability to cross the blood–brain barrier, and benefits in immunotherapy for glioblastoma were demonstrated.
Sonodynamic Therapy (SDT)	Ultrasonic activation of sensitive substances (Ce6, Mn^2+^ ions), causing ICD	Ce6/PTX Nbs	4T1 in vitro and in vivo	Inhibition of tumor growth and tumor metastasis formation was observed. The approach offers both imaging and therapeutic potential.
Nucleic Acids	Delivery of siRNA/miRNA or DNAzyme for PD-L1 silencing or immunostimulant production	AHA@MnP/siPD-L1	4T1 in vitro and in vivo	A strong anti-tumor effect and a high level of safety have been demonstrated.
		TT-LDCP	HCA-1, Hep3B, JHH-7 in vitro and in vivo	The need for an effective delivery system was highlighted. The designed system effectively inhibited the immune checkpoint and delivered the immunostimulatory cytokine.
		cGAMP-siPDL1@GalNPs	B16F10, 4T1 in vitro and in vivo	The anti-tumor effect of the primary tumor and distant tumors was demonstrated. Additional synergism with PDT has been demonstrated.
		dsDNA@DMONs	MC38, 4T1, Panc02, MDA-MB-231, A375, B16-F10 in vitro and in vivo	A dsDNA system was developed to induce IFN-I production inside the tumor, which indirectly activates the STING pathway. High therapeutic efficacy was demonstrated, with 51.0% inhibition of melanoma growth. In addition, the combination with anti-PD-L1 antibodies increased efficacy up to 96.7% regression.
Cuproptosis	Cu^2+^ release (+/− Zn^2+^) → mitochondrial stress → mtDNA → stronger cGAS-STING activation	CZP NPs	4T1 in vitro and in vivo	A combination of cuprotosis therapy, cGAS-STING activation, photothermal therapy, and immunotherapy was used. CZP NPs also increased the sensitivity of tumor cells to anti-PD-L1 treatment.
		CLDCu	B16F10 in vitro and in vivo	Inhaled nanoparticles have been developed to treat cancer metastases to the lungs.
Nanovaccines	Simultaneous delivery of cGAMP and antigen → presentation of neoantigen and activation of the immune system	LDH-cGAMP (RFA)	Hepa1-6 in vitro and in vivo	The inhibition of cancer growth and the establishment of long-term immunity have been observed.
		Acid-reactive polymer LNPs	B16-OVA, 4T1	Nanovaccines accumulated in lymph nodes and caused dendritic cell uptake and neoantigen release from the cytosol. The STING agonist activated the STING pathway in dendritic cells.

**Table 2:** Summary of selected nanoparticle-based STING agonist systems used in combination with PD-1/PD-L1 blockade. The table includes the active component, route of administration, and reported doses and dosages. Note that doses vary substantially across studies due to differences in nanoparticle composition, route of administration, and experimental model. Lower doses are typically used for highly potent agonists administered intratumorally, while higher doses are observed for systemic administration or for nanoparticles with lower payload efficiency [55,59,67,69,88,92,93].

Nanoparticle	Doses	Dosages (Days)
STING NPs	10 µg cGAMP i.t.	14, 17, 20
TPP-MMONs	10 mg/kg i.v.	0, 2, 4
NP2 (Pt(IV) + MK1775)	3 mg Pt kg^−1^, i.v.	0, 2, 4, 6, 8
G-M NPs	10 mg/kg intraperitoneal	1, 3, 5, 7, 9
cGAMP-siPDL1@GalNPs	25 μL; 15 μg of cGAMP per mouse and 15 μg of siPDL1/siNC per mouse i.t.	0
dsDNA@DMONs	25 µg/dose i.t.	3, 5, 7, 9
CZP NPs	10/20/30/40/50 mg/kg i.t.	2, 4, 6
CLDCu	10 mg/kg inhaled	3 times every 7 days

**Table 3:** Examples of current clinical trials evaluating combinations of STING agonists with checkpoint inhibitors (ICIs), including PD-1/PD-L1 blocking therapies, and using nanoparticles. Abbreviations: HNSCC—head and neck squamous cell cancer; TNBC—triple-negative breast cancer; ATC—anaplastic thyroid carcinoma; cSCC—cutaneous squamous cell carcinoma; ICI—immune checkpoint inhibitor; PULSAR—Personalized Ultra-fractionated Stereotactic Adaptive Radiotherapy (± combined or single; + combined).

Agonist/Platform	Formulation (NP/Free)	Trial/Status	Cancer Type	Combination	Key Note	Source
exoSTING (CDK-002)	Exosome-based NP	NCT04592484 Phase I/II Completed	HNSCC, TNBC, ATC, cSCC	Monotherapy	Focused on safety and pharmacodynamics; no extensive published results yet.	[144]
IMSA101	Free	NCT04020185 Phase I/IIa Completed	Different Solid Tumors	±ICI	Overall favorable safety profile in Phase I; transient cytokine-release-like events; RP2D established during escalation. Minimal sign of anticancer activity.	[145,149]
IMSA101	Free	NCT06601296 Phase II Recruiting	Metastatic Kidney Cancer	+nivolumab + PULSAR	Estimated study completion: 2028-10.	[150]
BI 1387446	Free	NCT04147234 Phase I Completed	Different Solid Tumors	±ezabenlimab (anti-PD-1)	Early data: good tolerability in phase I; biomarker analyses ongoing.	[146]
E7766	Free	NCT04144140 Phase I/Ib Terminated	Different Solid Tumors or Lymphomas	Monotherapy	Strong immune activation in phase I/Ib; AEs consistent with immune activation (fever, cytokine release).	[147]
Ulevostinag	Free	NCT03010176 Phase I/II Completed	HNSCC, TNBC	±pembrolizumab	In a small cohort, activity signal observed; most common AE: fever.	[148]
TAK-500	Free	NCT05070247 Phase I/II Terminated	Different Solid Tumors	±pembrolizumab	Clinical futility of TAK 500 met.	[151]
CRD3874-SI	Free	NCT06021626 Phase I Recruiting	Different Solid Tumors	Monotherapy	Estimated study completion: 2029-08.	[152]
